# Evaluation of fixed‐jaw IMRT and tangential partial‐VMAT radiotherapy plans for synchronous bilateral breast cancer irradiation based on a dosimetric study

**DOI:** 10.1002/acm2.12688

**Published:** 2019-09-04

**Authors:** Jiang‐Hua Huang, Xiu‐Xiu Wu, Xiao Lin, Jun‐Tian Shi, Yu‐Jia Ma, Song Duan, Xiao‐Bo Huang

**Affiliations:** ^1^ Guangdong Provincial Key Laboratory of Malignant Tumor Epigenetics and Gene Regulation, Medical Research Center Sun Yat‐Sen Memorial Hospital, Sun Yat‐Sen University Guangzhou China; ^2^ Department of Radiation Oncology Sun Yat‐Sen Memorial Hospital, Sun Yat‐Sen University Guangzhou China; ^3^ Department of Breast Tumor Center Sun Yat‐Sen Memorial Hospital, Sun Yat‐Sen University Guangzhou China

**Keywords:** dosimetric comparison, fixed‐jaw intensity‐modulated radiotherapy (F‐IMRT), synchronous bilateral breast cancer (SBBC), tangential partial volumetric modulated arc therapy (tP‐VMAT)

## Abstract

**Purpose:**

To investigate the fixed‐jaw intensity‐modulated radiotherapy (F‐IMRT) and tangential partial volumetric modulated arc therapy (tP‐VMAT) treatment plans for synchronous bilateral breast cancer (SBBC).

**Materials and method:**

Twelve SBBC patients with pTis‐2N0M0 stages who underwent whole‐breast irradiation after breast‐conserving surgery were planned with F‐IMRT and tP‐VMAT techniques prescribing 42.56 Gy (2.66 Gy*16f) to the breast. The F‐IMRT used 8‐12 jaw‐fixed tangential fields with single (sF‐IMRT) or two (F‐IMRT) isocenters located under the sternum or in the center of the left and right planning target volumes (PTVs), and tP‐VMAT used 4 tangential partial arcs with two isocenters located in the center of the left and right PTVs. Plan evaluation was based on dose‐volume histogram (DVH) analysis. Dosimetric parameters were calculated to evaluate plan quality; total monitor units (MUs), and the gamma analysis for patient‐specific quality assurance (QA) were also evaluated.

**Results:**

For PTVs, the three plans had similar D_mean_ and conformity index (CI) values. F‐IMRT showed a slightly better target coverage according to the V_100%_ values and demonstrated an obvious reduction in V_105%_ and D_max_ compared with the values observed for sF‐IMRT and tP‐VMAT. Compared with tP‐VMAT, sF‐IMRT was slightly better in terms of V_100%_, V_105%_ and D_max_. In addition, F‐IMRT achieved the best homogeneity index (HI) values for PTVs. Concerning healthy tissue, tP‐VMAT had an advantage in minimizing the high dose volume. The MUs of the tP‐VMAT plan were decreased approximately 1.45 and 1 times compared with the sF‐IMRT and F‐IMRT plans, respectively, and all plans passed QA. For the lungs, heart and liver, F‐IMRT achieved the smallest values in terms of D_mean_ and showed a significant difference compared with tP‐VMAT. Simultaneously, sF‐IMRT was also superior to tP‐VMAT. For the coronary artery, tP‐VMAT achieved the lowest D_mean_, while the value for F‐IMRT was 2.24% lower compared with sF‐IMRT. For all organs at risk (OARs), tP‐VMAT was superior at the high dose level. In contrast, sF‐IMRT and F‐IMRT were obviously superior at the low dose level. The sF‐IMRT and F‐IMRT plans showed consistent trends.

**Conclusion:**

All treatment plans for the provided techniques were of high quality and feasible for SBBC patients. However, we recommend F‐IMRT with a single isocenter as a priority technique because of the tremendous advantage of local hot spot control in PTVs and the reduced dose to OARs at low dose levels. When the irradiated dose to the lungs and heart exceed the clinical restriction, two isocenter F‐IMRT can be used to maximize OAR sparing. Additionally, tP‐VMAT can be adopted for improving cold spots in PTVs or high‐dose exposure to normal tissue when the interval between PTVs is narrow.

## 1 INTRODUCTION

Breast cancer is one of the most common types of cancer in females, and a significant number of women are diagnosed with breast cancer every year. Synchronous bilateral breast cancer (SBBC), defined as two or more malignant tumors occurring simultaneously in both breasts, is rare and complex, but the numbers of SBBC diagnoses have been showing an upward trend with an increase in the number of breast cancer cases. As for unilateral breast cancer (UBC), neoadjuvant or adjuvant chemotherapy, mastectomy or breast‐conserving surgery (BCS), and adjuvant radiotherapy are treatment options for SBBC. For most early stage UBCs, based on large randomized trials and meta‐analyses, there is convincing evidence showing that BCS plus radiotherapy is at least equivalent to mastectomy with respect to long‐term survival.^1^, ^2^, ^3^, ^4^, ^5^, ^6^, ^7^ Additionally, considering cosmetic and breast cancer‐specific survival probabilities, breast‐conservation treatment would be suggested, and standard treatment procedures include BCS followed by whole‐breast irradiation (WBI). However, no definite radiation therapy technique has yet been reported or established for SBBC. Compared with UBC irradiation, SBBC irradiation is more complex, with the concomitant involvement of both the lungs and heart and a wider distribution of treatment volume.

Three‐dimensional conformal radiotherapy (3D‐CRT) represents the most common approach for WBI. 3D‐CRT is generally delivered by two tangential fields for each breast and usually causes over/underdosage at field junctions and increased dose heterogeneity over the whole breast, especially in large‐breasted patients. Additionally, organs at risk (OARs) that lie in the same direction as the target cannot be fully protected.^8^, ^9^ In recent years, highly conformal radiation therapy techniques, such as intensity‐modulated radiation therapy (IMRT) and volumetric modulated arc therapy (VMAT), have been proposed to achieve the required target dose coverage while ensuring adequate normal tissue sparing. A few dosimetric studies have been conducted on IMRT, VMAT or helical tomotherapy for SBBC.^10^, ^11^, ^12^, ^13^, ^14^ Most of those studies employed a single isocenter located under the sternum. This approach can compress treatment time but is limited in clinical use, especially for obese patients. Moreover, in the aforementioned studies, how the treatment plans were executed was not specified, and more specific information regarding the treatment planning for bilateral breast cases was not provided.

In the present investigation, we designed fixed‐field IMRT (F‐IMRT) and tangential partial VMAT (tP‐VMAT) treatment plans for SBBC to identify an efficient method that can solve outstanding dose distribution problems and be applicable to various patients in the clinic.

## 2 MATERIALS AND METHODS

### 2.1 Patient selection and planning objectives

From February 2017 to March 2018, 12 SBBC patients with medical histories of ductal or lobular carcinoma were referred to our institution to receive adjuvant radiotherapy. All patients were diagnosed with stage pTis‐2N0M0 and underwent BCS; the median age was 45 (range, 31–64). CT scans were acquired with a thickness of 5 mm in free breathing mode; and the position was head‐first supine as the treatment position, with arms elevated. The scan range was from the sixth cervical to the second lumbar vertebra and included the entire lung volume and liver.

**Figure 1:**
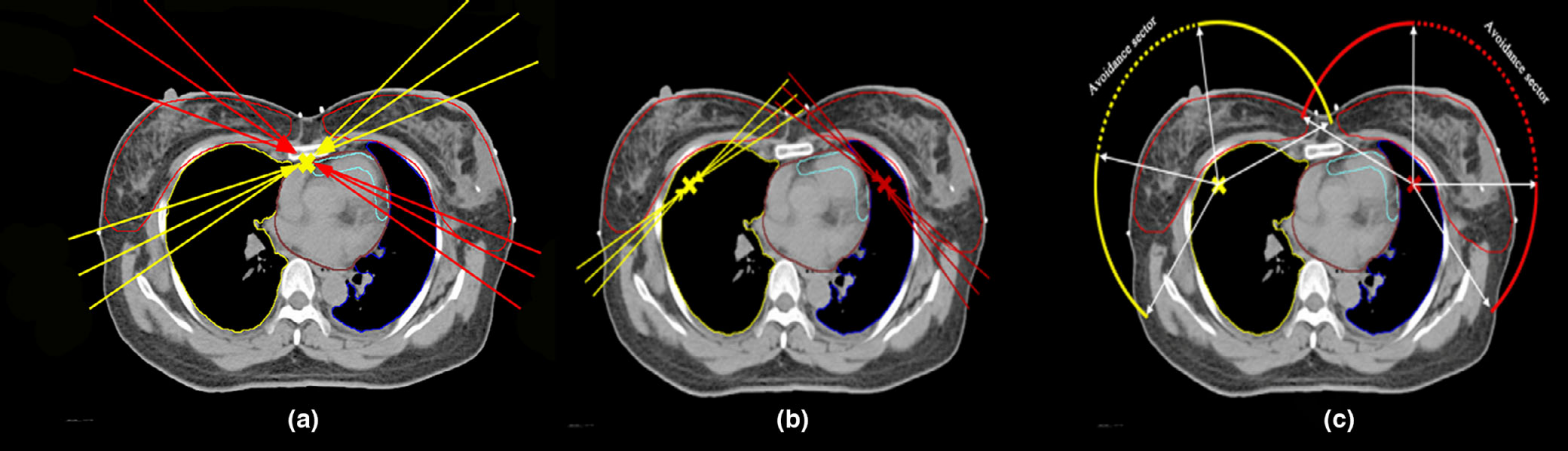
Beam arrangements and isocenter positions for (a) single fixed‐jaw intensity modulated radiotherapy (sF‐IMRT), (b) F‐IMRT, and (c) tangential partial volumetric modulated arc therapy. For F‐IMRT, six fixed‐jaw fields aimed to geometrically cover the left breast (red lines) and the other six (yellow lines) focused on the right breast. The interval angle for the ipsilateral fields was 6–10 degrees. For tangential partial VMAT, two partial arcs, rotating in opposite directions, aimed to geometrically cover primarily either the left (red line) or right (yellow line) breast. Dashed sectors were avoidance areas where the accelerator beam was off while the gantry was rotating.

All targets and structures were contoured by the same oncology physician according to ESTRO guidelines. The clinical target volumes (CTVs) were those encompassing the entire breast. The planning target volumes (PTVs) were obtained with an expansion of 8 mm in all directions from the CTVs and restricted to the skin cropping at 5 mm from the surface, excluding the ribs (Fig. 1). The mean volumes were as follows: PTV, 646.34 ± 164.88 cm^3^ (left), and 634.45 ± 146.67 cm^3^ (right). The main organs considered to be at risk were the lungs, heart, coronary artery area, and liver. The mean lung volumes were 915.66 ± 175.01 cm^3^ (left) and 1183.08 ± 210.28 cm^3^ (right); the mean heart volume was 540.58 ± 98.10 cm^3^. The mean coronary artery area volume was 67.34 ± 7.82 cm^3^, and the mean liver volume was 1270.71 ± 328.3 cm^3^.

As suggested in a previous study, appropriately dosed hypofractionated radiotherapy is safe and effective for patients with early breast cancer undergoing WBI and not only improves convenience but also may reduce acute pain, fatigue, and dermatitis.^15^, ^16^, ^17^, ^18^ We chose a regimen of 42.56 Gy in 16 fractions instead of a historical standard regimen (50 Gy in 25 fractions). Because we adopted a sequential boost scheme, the boost was not considered in this paper. Plans aimed to achieve 100% of the prescribed dose in 95% of PTVs and a maximum dose less than 105% of the prescription. No bolus was applied in any of the treatment plans. Because there is no definite treatment protocol for SBBC, the OAR dose constraints were established based on the results of previous SBBC studies^11^ and an attempt to maximize OAR sparing. The following conditions were established for OARs: for the lungs, mean dose < 15 Gy, V_5Gy_ < 70%, V_20Gy_ < 30%, and V_30Gy_ < 20%; for the heart, mean dose < 8 Gy and V_30Gy_ < 10%; for the coronary artery area, mean dose < 25 Gy and V_40Gy_ < 33%; and for the liver, V_30Gy_ < 30%.

### 2.2 Planning techniques

The plans were generated using the Varian Eclipse treatment planning system (TPS) version 13.5 by the same physicist. All plans were calculated by applying the data from a Varian Trilogy accelerator equipped with a 120 Millennium Multi‐leaf Collimator (MLC), which features a spatial resolution of 5 mm at the isocenter from the central 20 and 10 cm (spatial resolution of 10 mm) in the outer 2 × 10 cm, a maximum leaf speed of 2.5 cm/s, a leaf transmission factor of 1.5% and a dosimetric leaf gap of 0.16 cm. The Anisotropic Analytical Algorithm (AAA) algorithm was used for dose calculation, and calculation grid was set to 2.5 mm. The photon energy used was 6 MV, and a maximum dose rate of 600 MU/min for VMAT and a fixed dose rate of 600 MU/min for IMRT were applied.

#### 2.2.1 sF‐IMRT

The IMRT technique with multiple treatment fields has been reported to increase the low dose volumes in the ipsilateral and contralateral lung and heart.^10^, ^13^ In this study, we adopted the F‐IMRT technique to decrease heart and lungs irradiation without increasing the low dose volume.

**Figure 2:**
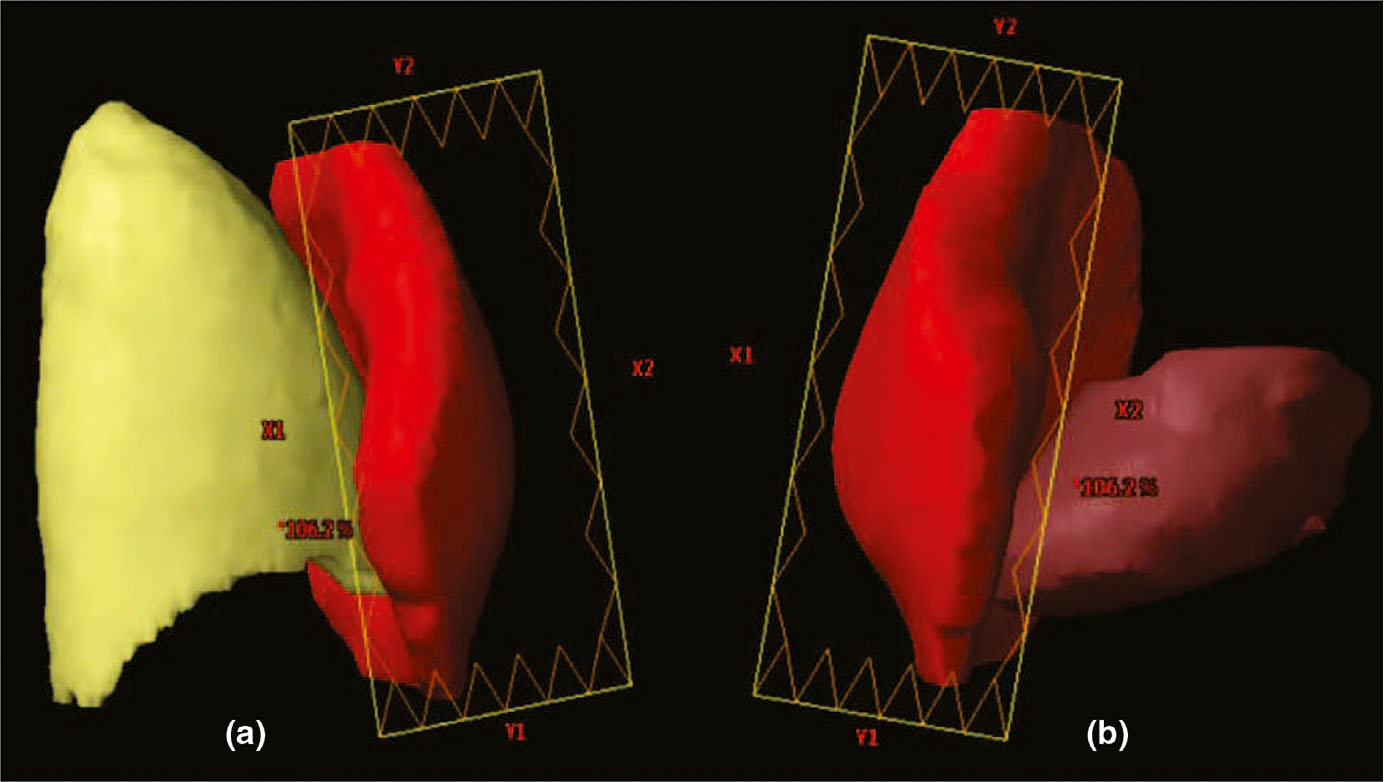
Example of fixed jaw use to reduce exposure of (a) right lung and (b) heart.

Initially, plans were optimized for a single isocenter approach, which was located medially under the sternum. Approximately 8–12 fixed‐jaw beams were used, and two or three pairs were similarly tangential beams for each target to avoid anterior and posterior entrances [Fig. 1(a)]. In the first step, we identified the inner tangent field in which the beam's eye view (BEV) of the target had the minimum projection. Then, taking this field as the starting point, the remaining one or two fields were identified in the clockwise direction (the counterclockwise direction was used for the right target), and each field was spaced 6–10 degrees. Finally, similar outer tangential fields were identified. All beams were coplanar beams, and the collimator angle was set parallel to the long axis of the focus targets. Fixed jaws have been used to maximize the OARs sparing in the outer tangential field. As shown in Fig. 2(a), the fixed X1 jaw can reduce lung exposure, and the shield target can be complemented by the opposite tangent fields. Similarly, the fixed X2 jaw in Fig. 2(b) can reduce heart irradiation.

#### 2.2.2 F‐IMRT

Different patients exhibit significant differences in breast shape and width in different parts of the breast. When the patient is obese, or the volume of the lungs is relatively small, high doses to the lungs and heart can result. When the irradiated dose to the lungs and heart exceeds the clinical restrictions, two isocenter F‐IMRT is used. In order to compare the sF‐IMRT approach, we used F‐IMRT technique for all 12 patients in this paper.

**Figure 3:**
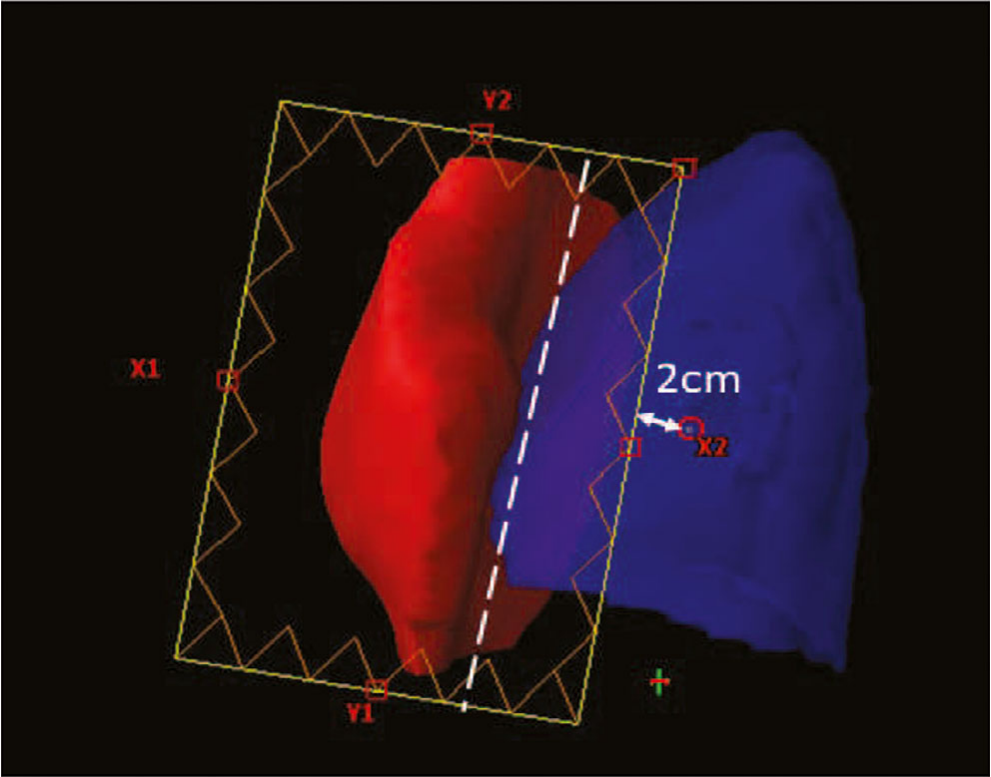
Example of fixed X2 jaw to reduce lung exposure. The X2 jaw can only exceed the central axis by 2 cm, and the white dotted line is where the X2 jaw is expected to be located.

There were two isocenters, each of which was located in the center of targets, one for the left PTV and one for the right PTV [Fig. 1(b)]. To facilitate clinical treatment, the two isocenters were located at the same level (only the patient's left‐right axis was different). Two isocenters rather than one are used for the following reasons. (a) The maximum distance the collimator jaw can extend over the central axis is 2 cm for the X jaws on a VARIAN machine. This limitation restricts the flexible use of the jaws in the single isocenter F‐IMRT method, which is more beneficial for sparing the lungs and heart, especially in SBBC or left‐sided whole‐breast irradiation. As body widths increase, the midline jaw of the outer tangential fields may not be fixed at the proximal target boundary. As shown in Fig. 3, the X2 jaw can only exceed the central axis 2 cm; the white dotted line is where the X2 jaw is expected to be located. (b) Moreover, to avoid collisions between the gantry and patient, the distance from the isocenter to the couch should be less than 22 cm. This value varies slightly depending on the immobilization device. For this reason, when body depths thicken, the isocenter must be lowered. This displacement could cause the field fluence generated by optimization processes to exceed 16.5 cm, which is the maximum limit for the VARIAN machine, and a calculation error would be generated in the dose calculation process. (c) When the curvature of the breast is large or the heart is very close to chest wall, two isocenter F‐IMRT can be used to further reduce OAR exposure. The rules for arranging treatment fields are the same as those previously established for sF‐IMRT.

Usually, two to three times optimizations can produce satisfactory results. With additional optimizations, the MU will be increased, which can be improved with a high smooth factor during the optimization phase. Considering the intrafraction motion of organs and interfraction setup errors, the tangential breast field in 3D‐CRT plans consistently and extensively irradiates the region outside the skin surface, which was defined by typically adding 2 cm to the anterior edge of the field. However, most commercially available TPSs assign a zero‐dose region outside the skin, which leads to the MLC close to the surface in IMRT plans. In this study, we used a skin flash tool to extend the fluence outside the surface and selected the appropriate cut range parameter and brush ceiling in BEV to extend the fluence uniformly by 2 cm outside the skin.

#### 2.2.3 tP‐VMAT

VMAT is a relatively new technique based on the simultaneous optimization of the MLC, gantry, and dose rate. Several studies have applied VMAT to UBC with varying results but with the same conclusions. Compared with tangential fields, VMAT achieves greater target coverage and homogeneity and reduces ipsilateral lung and heart doses in high dose volumes at the expense of increased low‐dose volumes.^19^, ^20^, ^21^ In previous dosimetric studies, full or partial arcs have been used for SBBC,^11^, ^12^, ^13^, ^14^ analogous to the use of VMAT for UBC, and the use of full or partial arcs consequently increased the irradiated volume. We introduced a restricted tangential partial VMAT technique to increase dose homogeneity and target coverage; furthermore, the radiation dose to the heart and lung can be decreased without increasing the low dose volume.

In the initial attempt, we planned the treatment using a single isocenter. However, the results did not reach our goal described in Section 2.A. Therefore, two isocenters were used in this method and were located in the same manner as in the two isocenter F‐IMRT plans. Four coplanar partial arcs were used in the present study: two for the left breast and two for the right breast, as shown in Fig. 1(c). For the left breast, those two arcs started from 150 to 300 degrees (one was clockwise, the other was counterclockwise), and 90 to 0 degrees was the avoidance sector; therefore, the accelerator only beamed on the 120 degree sector (150–90 degrees, 0–300 degrees). For the right breast, arcs started from 60 to 210 degrees (one was clockwise, the other was counterclockwise), and 350 to 280 degrees was the avoidance sector; the accelerator beamed on the 60–350 degree sector and the 280–210 degree sector. With respect to the heart, the avoidance sector for the left PTV was larger than that for the right PTV. To minimize the contribution of the tongue and groove effect during the gantry rotation, the TPS suggested a collimator angle fixed to 10–30 degrees instead of zero.^22^ In this study, the collimator angle was set parallel to the long axis of the focus targets.

Because no fluence map is generated in VMAT plans, the skin flash tool cannot be used to expand the fluence outside the skin. A 2‐cm‐thick bolus was added in the region of PTV outside the skin during optimization, but deliverable beams and dose calculations were performed without a bolus, as suggested in ICRU 62.^23^

In previous methods, all fields were simultaneously optimized to generate the desired dose distribution.

### 2.3 Evaluation tools

Plans were evaluated based on dose‐volume histogram (DVH) analysis. For PTV, the mean dose, D_max_, V_100%_ and V_105%_ (the volumes receiving at least 100% and 105% of the prescribed dose, respectively) were reported. The homogeneity index (HI) was calculated by the difference of D_2%_ and D_98%_ (dose received by 2% and 98% of the volume, respectively) to the prescribed dose.^24^ Low HI values indicate highly homogeneous target doses. The conformity index (CI) is defined as the ratio of the volume of the target covered by 98% of the prescribed dose to the total volume of PTV.^25^ CI values close to 1 indicate highly uniform coverage. To account for healthy tissue, we used the healthy tissue conformity index (HTCI) to evaluate excessive irradiation. HTCI is defined as the ratio of the volume of the target covered by 98% of the prescribed dose to the volume receiving the prescribed dose.

For the OARs, the mean dose and V_5Gy_, V_20Gy,_ and V_30Gy_ were compared for the lungs; the mean dose and V_30Gy_ were compared for the heart. The mean dose and V_40Gy_ were compared for the coronary artery area, and the mean dose and V_30Gy_ were compared for the liver.

Delivery parameters were recorded in terms of monitor units (MUs) for plans. Patient‐specific quality assurance (QA) for the IMRT and VMAT plans was performed using the Portal Dosimetry QA system with Varian Portal Vision (PV). The results were analyzed according to the gamma evaluation using 3% as the dose difference and 2 mm as the distance to the agreement with a 10% threshold. The gamma passing rate should be ≥95%.

An independent sample t‐test following a normality test (Shapiro‐Wilk) was used to compare the results, and the threshold for significance was *P* < 0.05.

## 3 RESULTS

**Figure 4:**
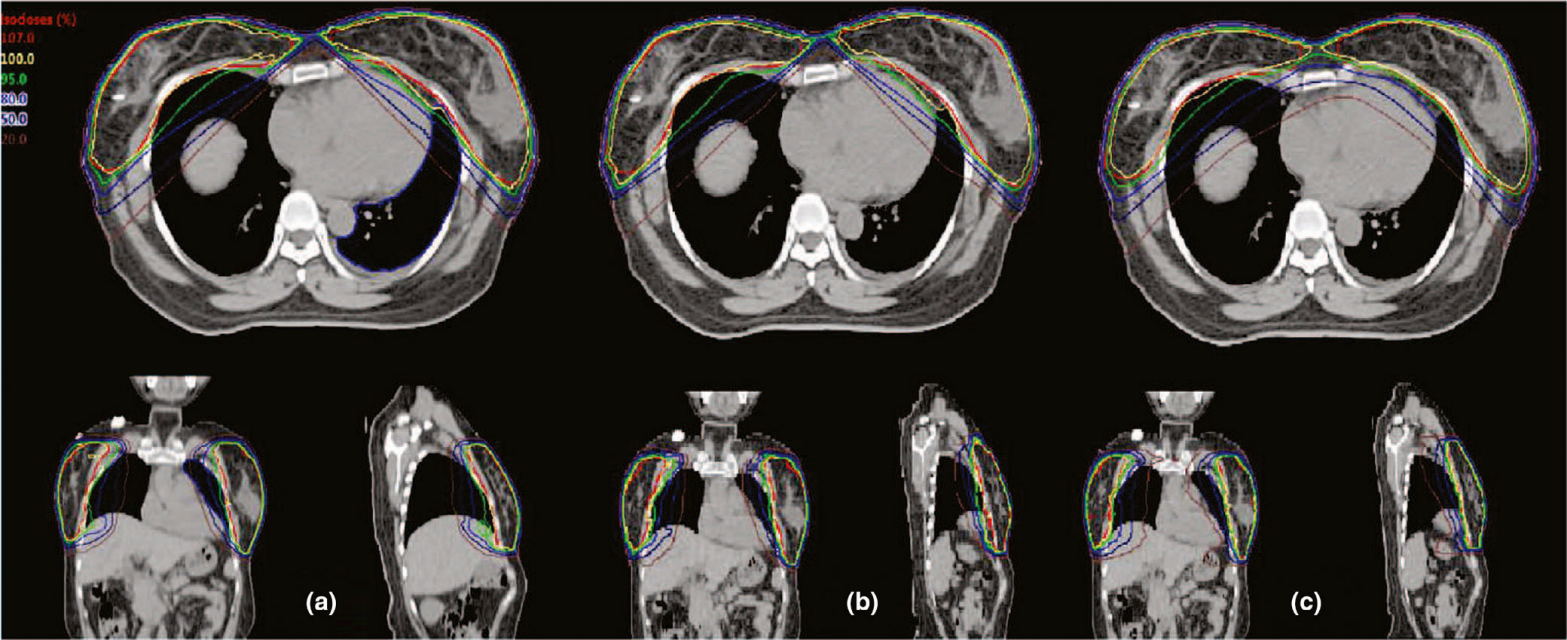
Case of isodose distribution on transverse, coronal, and sagittal views of (a) single fixed‐jaw intensity modulated radiotherapy (sF‐IMRT), (b) F‐IMRT, and (c) tangential partial volumetric modulated arc therapy for synchronous bilateral breast cancer.

**Figure 5:**
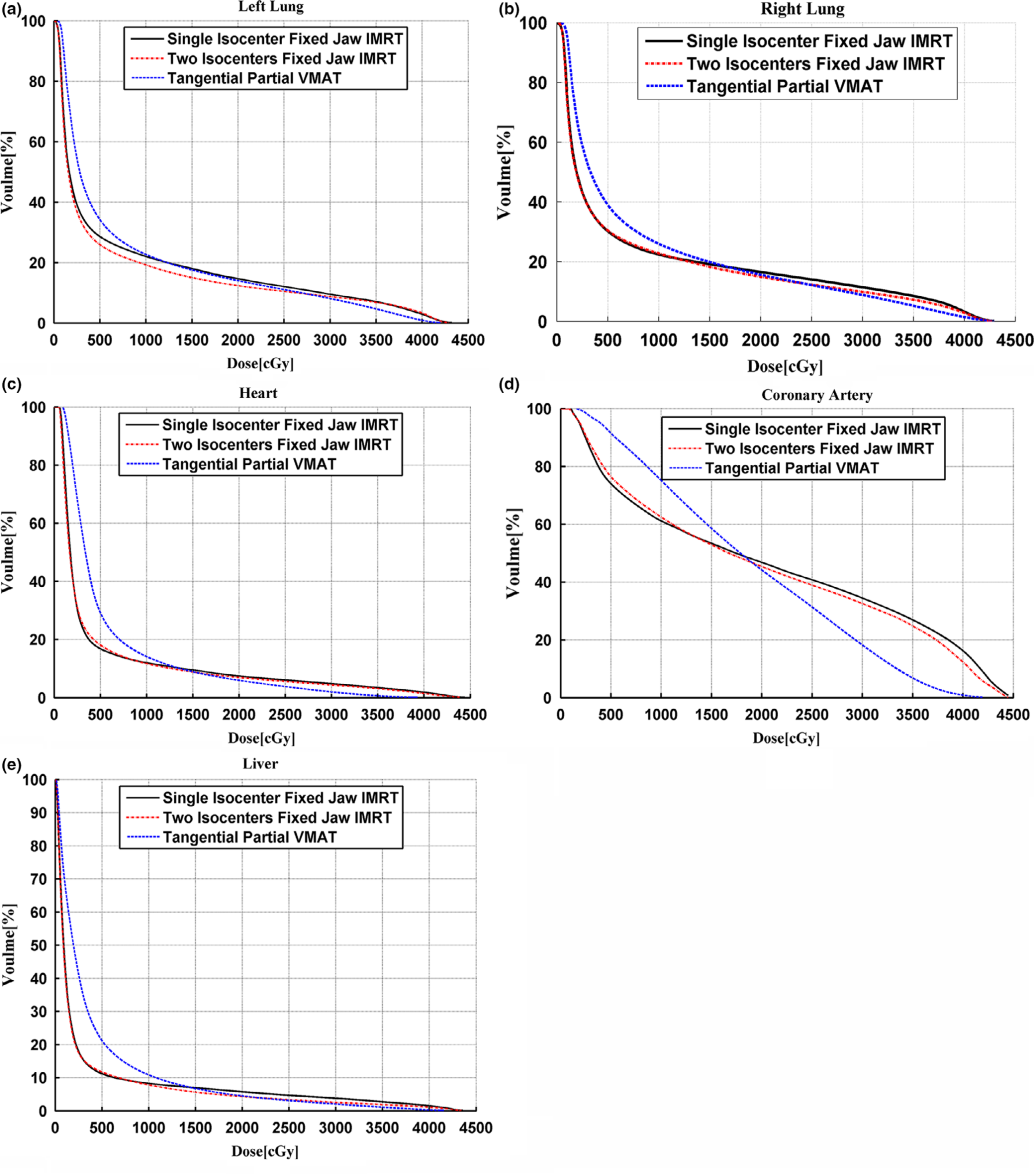
Mean dose‐volume histograms (averaged over the 12 patients) of the left (a) and right (b) lungs, heart (c), coronary artery area (d), and liver (e).

A case of SBBC isodose distributions with sF‐IMRT, F‐IMRT, and tP‐VMAT techniques is shown in Fig. 4 for the transverse, coronal, and sagittal planes. Figure 5 shows the mean DVHs for the lungs, heart, coronary artery area, and liver. The numerical DVH findings are summarized in Tables 1 and 2. All data in the tables are normally distributed, and the *P*‐values are shown.

**Table 1:** Comparison of PTVs coverage for single fixed‐jaw intensity modulated radiotherapy (sF‐IMRT), tangential partial volumetric modulated arc therapy (tP‐VMAT), and F‐IMRT.

	sF‐IMRT	F‐IMRT	tP‐VMAT	*P*‐values		
				sF‐IMRT vs tP‐VMAT	F‐IMRT vs tP‐VMAT	sF‐IMRT vs F‐IMRT
Left PTV						
D_mean_ (cGy)	4362.09 (17.67)	4366.14 (16.49)	4386.60 (12.57)	0.001	0.004	0.559
D_max_ (cGy)	4489.46 (35.79)	4468.30 (19.78)	4521.96 (14.69)	0.015	0.000	0.148
V_100%_	94.60 (2.22)	95.15 (0.60)	93.26 (1.7)	0.134	0.003	0.450
V_105%_	3.79 (5.15)	0.96 (1.52)	11.65 (7.03)	0.008	0.000	0.107
CI	0.983 (0.013)	0.985 (0.005)	0.980 (0.010)	0.610	0.212	0.703
HTCI	0.874 (0.584)	0.884 (0.051)	0.982 (0.033)	0.000	0.000	0.734
HI	0.066 (0.014)	0.061 (0.010)	0.077 (0.010)	0.040	0.001	0.444
Right PTV						
D_mean_ (cGy)	4365.72 (15.26)	4368.61 (17.63)	4387.2 (9.59)	0.001	0.006	0.546
D_max_ (cGy)	4494.34 (51.21)	4465.76 (24.91)	4514.13 (12.51)	0.239	0.000	0.143
V_100%_	94.87 (3.42)	95.70 (1.05)	94.42 (1.43)	0.376	0.002	0.450
V_105%_	5.29 (7.97)	1.03 (1.88)	9.21 (5.56)	0.198	0.000	0.114
CI	0.983 (0.018)	0.989 (0.005)	0.983 (0.007)	0.998	0.024	0.315
HTCI	0.757 (0.055)	0.805 (0.075)	0.884 (0.022)	0.000	0.003	0.127
HI	0.070 (0.026)	0.057 (0.009)	0.073 (0.007)	0.686	0.000	0.153
MU	2742.55 (604.93)	2157.91 (371.51)	953.64 (66.16)	0.000	0.000	0.014
QA (%)	97.20 (0.98)	97.35 (1.02)	98.51 (0.73)	0.534	0.581	0.612

**Table 2:** Comparison of organs at risks dose for single fixed‐jaw intensity modulated radiotherapy (sF‐IMRT), F‐IMRT, and tangential partial volumetric modulated arc therapy (tP‐VMAT).

	sF‐IMRT	F‐IMRT	tP‐VMAT	*P*‐values		
				sF‐IMRT vs tP‐VMAT	F‐IMRT vs tP‐VMAT	sF‐IMRT vs F‐IMRT
Left lung						
D_mean_ (cGy)	748.50 (122.54)	676.70 (112.26)	785.52 (37.94)	0.358	0.006	0.110
V_30Gy_ (%)	9.85 (2.86)	8.76 (2.06)	8.18 (1.05)	0.094	0.415	0.196
V_20Gy_ (%)	14.25 (2.81)	12.35 (2.47)	14.23 (1.50)	0.978	0.043	0.077
V_5Gy_ (%)	29.54 (4.69)	25.90 (4.58)	34.23 (1.54)	0.008	0.000	0.050
Right lung						
D_mean_ (cGy)	829.13 (115.31)	773.82 (93.73)	876.19 (75.43)	0.273	0.011	0.095
V_30Gy_ (%)	11.85 (2.92)	9.84(2.29)	8.99 (1.62)	0.012	0.327	0.068
V_20Gy_ (%)	16.80 (2.53)	14.89(2.23)	16.02 (2.13)	0.446	0.236	0.045
V_5Gy_ (%)	31.63 (5.66)	30.41(3.92)	39.53 (4.34)	0.002	0.000	0.398
Heart						
D_mean_ (cGy)	505.68 (164.10)	479.35 (125.54)	580.59 (87.12)	0.201	0.040	0.634
V_30Gy_ (%)	4.59 (2.47)	4.38 (2.20)	2.02 (1.10)	0.007	0.005	0.767
Coronary						
D_mean_ (cGy)	2024.85 (576.93)	1980.44 (519.10)	1883.26 (335.65)	0.492	0.608	0.792
V_40Gy_ (%)	15.12 (8.11)	12.30 (7.82)	0.87 (1.49)	0.000	0.000	0.393
Liver						
D_mean_ (cGy)	357.85 (167.79)	307.88 (123.71)	439.71 (125.57)	0.211	0.022	0.391
V_30Gy_ (%)	3.80 (2.70)	2.57 (1.67)	1.88 (1.25)	0.109	0.307	0.200

### 3.1 PTV dose distribution

The comparison data in Table 1 are reported for the left and right PTVs. In general, the sF‐IMRT, F‐IMRT and tP‐VMAT plans achieved similar mean values for PTVs. The mean values of the tP‐VMAT plans were 4386.60 ± 12.57 cGy for the left PTVs, an approximately 0.56% and 0.47% higher dose than that of sF‐IMRT (*P* = 0.004) and F‐IMRT (*P* = 0.001), respectively, and 4387.2 ± 9.59 cGy for the right PTVs, an approximately 0.49% and 0.42% higher dose than that of sF‐IMRT (*P* = 0.006) and F‐IMRT (*P* = 0.001), respectively.

PTV coverage with the prescribed dose in F‐IMRT was the best, with values of 95.15 ± 0.60% for left PTVs and 95.70 ± 1.05% for right PTVs, while sF‐IMRT and tP‐VMAT presented a minor violation. Furthermore, a better high‐dose control was achieved for F‐IMRT compared with sF‐IMRT and tP‐VMAT according to the D_max_ and V_105%_ values in Table 1. For the left PTVs, V_105%_ was 0.96 ± 1.52% for F‐IMRT, 3.79 ± 5.15% for sF‐IMRT and 11.65 ± 7.03% for tP‐VMAT. For the right PTVs, V_105%_ was 1.03 ± 1.88% for F‐IMRT, 5.29 ± 7.97% for sF‐IMRT, and 9.21 ± 5.56% for tP‐VAMT. Although the sF‐IMRT and tP‐VMAT in the high‐dose control were not as favorable as the F‐IMRT plans, the D_max_ value was restricted to 107% of the prescribed dos at 4489.46 ± 35.79 cGy and 4521.96 ± 14.69 cGy, respectively, for left PTVs, 4494.34 ± 51.21 cGy and 4514.13 ± 12.51 cGy, respectively, for right PTVs. The sF‐IMRT demonstrated a slight advantage compared with tP‐VMAT. The HI values for three plans were <0.08 (closer to 0), while F‐IMRT showed better homogeneity for both PTVs according to the lower HI values. However, tP‐VMAT could greatly reduce high‐dose exposure to healthy tissue according to the higher HTCI values.

Table 1 also shows the average MU per fraction for the different plans, and the difference in MUs was significant. As tP‐VMAT plans obtained the fewest number of MUs, the delivery time was notably reduced. The MU ratio between F‐IMRT and tP‐VMAT was 2.04. Simultaneously, the MU ratio between sF‐IMRT and tP‐VMAT was 2.44. All QA results were above the criterion, which indicated those plans were clinically acceptable.

The data for the 12 patients showed that the three planning methods were eligible. Comparatively, the F‐IMRT plan yielded the best results. During the planning process, F‐IMRT reached the stated goals faster and more consistently.

### 3.2 OAR dose distribution

Table 2 shows a statistical comparison of the OARs for plans; all values met the dose restriction conditions. The results presented in Fig. 5 are consistent with those in Table 2. The mean values for the lungs, heart, and liver indicated in Table 2 were significantly different between the F‐IMRT and tP‐VMAT; however, the other two groups showed no significant differences. The DVHs in Fig. 5 indicate that the values for tP‐VMAT were distinctly lower than those for sF‐IMRT and F‐IMRT at high dose levels and were higher at low dose levels. Obviously, F‐IMRT had similar DVHs with sF‐IMRT.

Bilateral lung protection was our concern. All mean values for both lungs under the plans were managed under 9 Gy, and F‐IMRT plans protected lungs best with the lowest mean values (676.70 ± 112.26 cGy for the left lungs and 773.82 ± 93.73 cGy for the right lungs). The mean values for F‐IMRT were approximately 16.08% (left lung) and 13.23% (right lung) lower than those for tP‐VMAT. Simultaneously, the observed differences were significant. Specifically, F‐IMRT proved to be slightly superior to tP‐VMAT at low dose levels (e.g., V_5_, V_20_), and at higher dose levels, tP‐VMAT was better than F‐IMRT, as shown in Figs. 5(a) and 5(b). Compared with sF‐IMRT, the mean values were 10.61% (left lungs) and 7.15% (right lungs) lower for F‐IMRT; however, the difference was not significant (p> 0.05).

For the heart, all plans yielded ideal results, in which the mean values were controlled at 6 Gy. However, F‐IMRT showed a lower D_mean_ of 1 Gy for the heart compared with tP‐VMAT, and the difference was significant. F‐IMRT was better at low dose levels, whereas tP‐VMAT was superior to F‐IMRT at high dose levels. For sF‐IMRT, the difference with F‐IMRT was small and without significance; as shown in Fig. 5c, the two DVHs had a similar trend.

For the coronary artery area, tP‐VMAT achieved a D_mean_ of 1883.26 ± 335.65 cGy, which was nearly 7.52% and 5.16% less than that achieved by sF‐IMRT and F‐IMRT. A distinct reduction in volumes at high dose levels (e.g., V_30Gy_ and V_40Gy_) was observed for tP‐VMAT, and the difference in terms of V_40Gy_ was statistically significant. However, there was no substantial difference between sF‐IMRT and F‐IMRT.

For the liver, F‐IMRT achieved the lowest mean values (307.88 ± 123.71 cGy), followed by sF‐IMRT (357.85 ± 167.79 cGy) and tP‐VMAT (439.71 ± 125.57 cGy). The difference was significant only between F‐IMRT and tP‐VMAT. At low dose levels, sF‐IMRT and F‐IMRT was superior to tP‐VMAT, except for a slightly higher contribution at high dose levels.

## 4 DISCUSSION

SBBC involves a tremendous target volume and is closer to OARs, such as the hearts and lungs. Kim et al. used 3D‐CRT for SBBC.^13^ The PTV coverage V_95%_ in that study was 93.65 ± 2.81% for the left PTVs and 93.48 ± 2.74% for the right PTVs, which were lower than V_100%_ in the present study. Furthermore, a better high‐dose control V_105%_ was achieved, as shown in Table 1, compared with 16.07 ± 11.57% for the left PTVs, 14.88 ± 9.64% for the right PTVs in that literature. For OARs, the V_5_ and V_20_ were 35.71 ± 9.18% and 22.65 ± 8.67% for the left lung, 38.74 ± 7.23% and 22.51 ± 6.36% for right lung; in terms of the heart, the mean value was 8.18 ± 3.06 Gy. These data are higher than those presented in Table 2. As we known, 3D‐CRT is typically affected by the risk of over/underdosage at the field junctions, increased dose heterogeneity over the targets and large portions of heart and lungs that cannot be dosimetrically spared. The purpose of this study was to provide an alternative protocol for SBBC irradiation treatment with highly conformal RT while assuring adequate normal tissue sparing. Although several studies on SBBC have been conducted, the planning details have not been sufficient. The sF‐IMRT, F‐IMRT, and tP‐VMAT methods were suggested in this study to improve target coverage and subsequent disease control while sparing normal tissue and reducing patient toxicity.

In present study, the patients were treated in the supine position. Several studies have shown that the ipsilateral lung dose is significantly decreased when the patient is treated in the prone position compared with supine, but no significant differences were detected with regard to the dose to the heart.^26^, ^27^ However, the prone treatment position has been proposed for patients with large or pendulous breasts to reduce the dose to the heart and the ipsilateral lung.^28^ To further avoid the irradiation of heart, the deep inspiration breath‐hold (DIBH) technique has been suggested for WBI, especially for left‐side breast cancer.^29^ Unfortunately, there are a number of patients that cannot hold breath for long times or are having difficulties to manage the breath‐hold techniques. Furthermore, some institutions do not have breath‐hold devices or sufficient time for every breast cancer patient to use the DIBH technique, which usually occurs in large‐workload units.

In present study, we attempted to use a single isocenter to achieve a homogeneous dose coverage, simultaneously reducing doses to OARs. Most patients can obtain satisfactory results, except for the obese. When the X Jaw needs to extend over the central axis above 2 cm or the distance from isocenter located under the sternum to the couch exceeds 22 cm, there is a risk of collision between the gantry and patient; the two isocenter F‐IMRT can solve this challenge. In fact, not every patient will experience either of the two above‐mentioned conditions; therefore, a single isocenter located under the sternum is the first choice for SBBC with the same field management procedure. VMAT uses continuous variation of the instantaneous dose rate, MLC leaf positions and gantry rotational speed to optimize the dose distribution. The number and composition of arcs have serious effects on the dose distribution. Nicolini et al. used two arcs of 360° each; the first arc, rotating clockwise, was incident primarily on the right breast, and the second arc, rotating counterclockwise, was incident on the left breast.^11^ In another study, two half‐arcs (180 degrees) consisted of a rotating beam on each breast.^13^ Those continued arcs increased the low‐dose volume to the lungs and heart compared with the volume observed in the tangential field technique. Qiu et al. used a modified partial arc in which a portion of the arc was blocked to minimize the normal structure dose for partial breast irradiation.^30^ Another study used two tangential dual arcs of 50‐60° to decrease the low‐dose volume for the ipsilateral lung and heart for left WBI.^31^ In the present study, four tangential partial arcs were used: two for the left breast and two for the right breast. The first step was to determine the partial arc range based on the individual PTV location. Then, a shield area was selected within the arc to avoid angles directed toward the heart and lungs. The avoidance sectors were defined before the optimization procedure. Usually, the avoidance sectors in the left breast are larger than those in the right breast, owing to the heart and coronary artery area.

The three techniques selected for this investigation were meticulously designed with tangential fields to improve dose coverage and decrease the radiation exposure of normal tissue. The data presented in the tables suggest that three techniques can obtain satisfactory results as described in section 2.A for the treatment of SBBC. As long‐term breast pain, cosmesis, and quality of life highly correlate with moist desquamation and severe acute pain during radiation treatment, it is justified to optimize dose homogeneity to prevent acute side effects, particularly moist desquamation.^32^ The 2018 ASTRO Guideline recommendation for dose homogeneity for hypofractionated whole‐breast irradiation (HF‐WBI) is that the volume of breast tissue receiving 105% of the prescribed dose should be minimized, regardless of dose fractionation.^33^ According to the V_105%_ values in Table 1, F‐IMRT showed the smallest dose distribution, with only approximately 1% of both PTVs, which is a great advantage compared with sF‐IMRT and tP‐VMAT. Additionally, the F‐IMRT plan was superior to the other plans with regard to HI and CI.

However, hot spots out of target and cold spots in PTVs were challenges for sF‐IMRT and F‐IMRT because the number of tangential fields was limited, and the dose intensity modulation was limited from the tangential directions. Thus, hot spots out of target could often be found, especially above the sternum when the interval between the left and right PTVs was narrow. Based on this study, we found that when the interval distance was smaller than 2.5 cm, hot spots could only be eliminated at the expense of PTV coverage in sF‐IMRT and F‐IMRT plans. Simultaneously, cold spots in PTVs adjacent to the sternum were also a distinct disadvantage due to tight high‐dose control in PTVs and strict normal tissue avoidance, especially when the tumor bed happened to be near the inner tangential field in the PTV area. Additionally, among BRCA carrier patients treated for early breast cancer, prophylactic irradiation to the contralateral breast to reduce the risk of subsequent contralateral breast cancer is gradually gaining wide acceptance.^34^ Because there is no accurate tumor bed in the contralateral breast, the cold spots that appear in the breast adjacent to the sternum may influence this prophylactic irradiation effect. tP‐VMAT further reduced the presence of hot spots out of target and improved cold spots in targets with multiple tangential partial arcs and simultaneous modulation of the dose rate. Therefore, in this situation, the dose coverage of tP‐VMAT is superior to that of sF‐IMRT and F‐IMRT.

Regarding OARs and normal tissue, the reduction of the high‐dose region decreases the incidence of acute and late toxicities, such as acute radiodermatitis, symptomatic radiation pneumonitis and skin fibrosis. Moreover, the low‐dose region within OARs may decrease the incidence of second primary malignant tumors induced by radiotherapy.^35^ The tP‐VMAT method can increase the volume of tissue exposed to low doses of radiation and thus increase the risk for radiation‐induced carcinogenesis. The technique has also been shown to decrease the volume of tissue exposed to high doses of radiation, which could exert a beneficial impact on the risk for radiation‐induced sarcoma (RIS). However, the overall risk for RIS is small compared with the potential benefit of radiotherapy.^36^

To verify the efficiency of each technique, we compared MUs. The MUs optimized by tP‐VMAT were far less than those of sF‐IMRT and F‐IMRT. Because of the reduced number of MUs in tP‐VMAT, the delivery time was reduced, which should not be neglected in clinical treatment.

## 5 CONCLUSION

In our research, the sF‐IMRT, F‐IMRT and tP‐VMAT techniques could produce treatment plans of high quality for SBBC patients. As sF‐IMRT is designed with one isocenter, it can be performed conveniently in clinical treatment with a decreased setup time. F‐IMRT plans showed better physical dose distribution in PTVs and superior protection in OARs. tP‐VMAT resulted in an obvious reduction of high‐dose exposure for normal tissue and OARs and was superior in improving hot spots out of target and cold spots in target. In summary, it is difficult to determine which technique is appropriate for all patients with SBBC, but we recommend sF‐IMRT as a priority technique. F‐IMRT could be adopted when a patient is obese, and tP‐VMAT is more appropriate for improving inadmissible cold spots in PTVs or overrunning high‐dose exposure to normal tissue when the interval between PTVs is narrow. Further studies involving additional patient data and improved techniques are needed.

## CONFLICT OF INTEREST

The authors have no relevant conflict of interest to disclose.
